# Indian nurses’ beliefs on physical activity promotion practices for cancer survivors in a tertiary care hospital—a cross-sectional survey

**DOI:** 10.7717/peerj.13348

**Published:** 2022-05-23

**Authors:** Hritika D. Pai, Stephen Rajan Samuel, K. Vijaya Kumar, Namrata S. Chauhan, Charu Eapen, Alicia Olsen, Justin W.L. Keogh

**Affiliations:** 1 Department of Physiotherapy, Kasturba Medical College, Mangalore, Manipal Academy of Higher Education, Manipal, Karnataka, India; 2 Faculty of Health Sciences and Medicine, Bond University, Gold Coast, Australia; 3 Southern Cross University, National Centre for Naturopathic Medicine, Lismore, New South Wales, Australia; 4 Human Potential Centre, AUT University, Auckland, New Zealand; 5 Cluster of Health Improvement, University of the Sunshine Coast, Sunshine Coast, Australia

**Keywords:** Cancer, Nurses, Survivorship, Exercise, Neoplasm

## Abstract

**Purpose:**

To describe the physical activity (PA) promotion practices, beliefs, and barriers of Indian nurses working with cancer survivors, and to gain preliminary insights into how their educational qualification might affect PA promotion practices.

**Methods:**

A validated questionnaire was used to obtain the data (*N* = 388). Sub-group comparisons were performed based on nursing qualification *i.e.*, Bachelor of Science in Nursing (BSc) and General Nursing and Midwifery (GNM) using Mann-Whitney U test and chi square analysis for continuous and categorical variables, respectively.

**Results:**

The nurses believed that oncologists (47%) followed by physiotherapists (28.9%) were primarily responsible for providing information regarding PA to cancer survivors. The most common period in which the nurses’ promoted PA was post treatment (31.7%), although very few nurses (13.3%) promoted PA across more than one of the three treatment periods. Nurses felt that PA had many benefits for cancer survivors; improved mental health (87.7%) and HRQoL (81.1%). Lack of knowledge (42.2%) and lack of time (41.6%) were the most frequently cited barriers. The comparisons based on educational qualification did not typically reveal many significant differences.

**Conclusion:**

Indian nurses both BSc and GNM qualified, wish to promote PA to cancer survivors despite numerous barriers, across various stages of treatment and believe PA is beneficial to the survivors in the process of recovery. Overcoming these barriers might aid in better promotion of PA to cancer survivors.

**Implication for cancer survivors:**

Nurses working in a tertiary care hospital in India are willing to promote PA amongst cancer survivors but require more training and support in this area of practice.

## Introduction

India, like many other countries, has a very high incidence of non-communicable disease (NCD) related death (World Health Organization, 2019). Evidence suggests that the projected incidence of patients with cancer in India for the year 2020 was approximated to 94.1 per 100,000 among males and 103.6 per 100,000 among females (Mathur et al., 2020). Planned cancer-directed treatment across the cancer population in India comprises of surgery, radiotherapy, chemotherapy, systemic therapy, and multimodality treatment (a combination of surgery and/or radiotherapy and/or systemic therapy). Cancer treatments have deleterious effects on the survivors in terms of performance of daily activities and health-related quality of life, due to adverse events such as nausea, vomiting, loss of appetite, fatigue, hair loss, diarrhea, insomnia, and associated infections. There are also detrimental effects seen in the patients’ body composition (muscle, bone and fat mass), and physical function over time, thus posing an increased threat for the development of cardiovascular and orthopedic complications (Karvinen et al., 2012; Karvinen et al., 2017; Kapoor et al., 2015; Hayes et al., 2019; Indian Nursing Council, 2021).

Regular PA provides benefits including improved body composition, muscle strength, muscular and aerobic endurance, significant reductions in depression and fatigue as well as improved health-related quality of life and survival rates for a range of cancer survivors (Kintzel et al., 2008; Leahy et al., 2013a). As a result, the American Cancer Society (ACS) general guidelines suggest that cancer survivors should perform 30 mins of moderate to vigorous PA at least 5 days per week, with 45–60 min of intentional PA per day (Thorsen et al., 2008). The promotion and/or prescription of a well-established PA regimen is essential for cancer survivors to reduce the severity of cancer symptoms and aiding the recovery process (Hayes et al., 2019). Health professionals *i.e.*, the oncologists, physical therapists, exercise physiologists as well as the nursing staff are in a position to actively promote PA to cancer survivors (Kintzel et al., 2008; Leahy et al., 2013a).

Nurses work as navigators to maintain the continuity of cancer treatment and bring about positive survivor outcomes (McMullen, 2013). Further, nurses may be the most influential healthcare professional group as they typically have more frequent contact with their patients, meaning they can better cater to the patients’ immediate needs and provide appropriate counselling more so than the rest of the oncology team (Leahy et al., 2013b). This suggests that nurses may have unique opportunities to aid the cancer survivors recovery, and to discuss with the survivors their primary barriers and facilitators in the performance of PA (Keogh et al., 2017b). However, the nursing staff and the other health professionals who wish to create awareness about the importance of PA among their patients face barriers because it is not their primary area of specialization, and they may lack adequate evidence-based information to provide to their patients. Additionally, nurses themselves may be unaware of the importance of PA and its beneficial effects among cancer survivors (Oefelein et al., 2002; World Health Organization, 2019). Hence, this cross-sectional survey aimed at identifying the beliefs of the Indian nurses regarding the importance of PA amongst cancer survivors. In addition, this study also aimed to gain further insight into the barriers faced by Indian nurses’ who wish to promote PA to cancer survivors in a tertiary care hospital.

## Methods

### Study design

This observational study was conducted to gain preliminary insights into the PA promotion beliefs and practices of Indian nurses. This is important because India is a country with a very large cancer population but no documented peer-reviewed literature on PA promotion practices of nurses to their patients. The questionnaire used in the study was virtually identical to that used in an Australasian study of cancer nurses (Keogh et al., 2017b), with the questions based on the theories of planned behavior and social cognitive theory. To minimize response bias, questions about dietary promotion practices were also included, so that nurses who promote improved diet but not PA to their cancer patients are more likely to participate and provide honest responses in this study. While online surveys are becoming more common in many countries for assessing health promotion behaviors of health professionals (Puhringer et al., 2015; Physical Activity Guidelines Resources, 2021), it was felt that a greater response rate would be obtained with physical (paper) questionnaires, as initial consultations with nursing representatives indicated a preference for physical (paper) questionnaires over the online version of the questionnaires.

### Participants and procedures

Three hundred eighty-eight nursing staff from a tertiary care hospital in the Dakshin Kannada district, Karnataka State, India were invited to participate in this study. In India, there are two common pathways to a career in nursing, namely BSc (Bachelor of Science in Nursing) and GNM (General Nursing and Midwifery) (Indian Nursing Council, 2021). The BSc nursing staff successfully complete high school subjects in physics, chemistry, and biology prior to beginning their university degree, which takes 4 years. The nursing staff who do not opt for physics, chemistry, and biology as high school subjects but instead successfully completed mathematics subjects, can enroll in a General Nursing and Midwifery (GNM) degree for 3 years. The estimation of sample size was carried out using the estimation of proportion method, and the sampling technique incorporated for the cross sectional survey was a convenience sampling method.

The study protocol and the survey questionnaire were approved by the Scientific Committee and the Institutional Ethics Committee, Kasturba Medical College, Mangalore, Approval No: IEC KMC MLR 02-2021/59). After obtaining ethical approval, the medical superintendent, and the nursing superintendent of the respective hospital were contacted to provide gatekeeper approval. The nursing superintendents of the two wings called for a meeting with the respective ward in-charges (supervisors) *i.e.*, intensive care units, emergency unit, general wards, COVID ICU and COVID wards, paediatric intensive care units, wards and post-operative units. In this meeting, each of the supervisors were provided information about the study and the supervisors volunteered to be the point of contact for the nurses to have access to the questionnaire and return them to the investigators. Due to odd working hours, 12 h shifts and frequent rotation’s in COVID wards and ICUs, the nursing superintendents advised that we give the sets of questionnaires and informed consent forms to the supervisors. The informed consent forms provided the participants with the email address and phone numbers of the investigators if they had any questions regarding the questionnaires. The investigators followed up on alternative days with the supervisors. If the supervisors or potential participants had any questions about the project and/or questionnaire, these were sorted through telephone conversations with the researchers. As is quite standard for surveys, there were some missing values for different questions. As a result, the total number of respondents for each question is provided in the tables so to provide the reader some insight into the number of respondents for each question.

### Instrument

The survey questionnaire that was used in this study was based on the framework described in a previous study conducted among Australasian nurses (Keogh et al., 2017b). The questionnaire consisted of three sections *i.e.*, demographics of the nurses, the prevalence of health promotion in their respective hospitals, and the nurses’ motivational aspects associated with PA among cancer survivors. The questions about the demographics of the nurses such as, age, gender, the highest level of professional qualification, the location of the hospital, and the nurses’ specializations were incorporated along with questions that addressed the nurses’ current level of PA.

Inclusion of multiple-choice questions in the survey were done to obtain information on the promotion of PA practices among the nurses *e.g.*, their perceptions on which professional group is responsible for the promotion of PA for patients with cancer, and their attitude towards the promotion of PA to their patients with cancer. The promotion of PA to cancer survivors during different stages of cancer (pre-, during-, and post-treatment) was also assessed using multiple-choice items.

The nurses’ beliefs regarding the beneficial effects of PA among cancer survivors were assessed on a Likert scale ranging from 1 to 4 *i.e.*, 1—strongly disagree, 2—disagree, 3—agree, 4—strongly agree for nine different factors which are: (1) improves health-related quality of life, (2) improves weight management (3) improves fatigue level, (4) improves mental health, (5) improves activities of daily living, (6) reduces the risk of cancer recurrence, (7) reduces the risk of other chronic diseases, (8) reduces tumor-specific comorbidities and, (9) no benefits. The nurses were also asked whether the cancer survivors are generally uninterested in PA, and their opinion on whether the promotion of PA to cancer survivors is entirely up to them. In addition, information regarding the beliefs of their fellow nurses on the promotion of PA among cancer survivors and if there is a strong base suggesting the promotion of PA among cancer survivors.

In addition, information on the commonly cited barriers the nurses face in the promotion of PA to cancer survivors was also included in the questionnaire. The listed barriers included a lack of time, risk to the patient, lack of adequate support structure, lack of knowledge, lack of expertise, the promotion of PA is not their job, or that they do not have barriers in the promotion of the PA.

As there exist many differences between the healthcare system and language in India and Australasia, a number of minor modifications to the wording of the questionnaire items were made by the researchers with respect to the original survey (Keogh et al., 2017b). The slightly revised questionnaire was then provided to a sample of 10 nursing staff, as well experts from the fields of oncology (*n* = 2), physical therapy (*n* = 5), and nursing (*n* = 1). The respondents were asked open-ended questions about each questionnaire item and what their corresponding responses meant. Their responses were marked on a 3-point scale (agree, neutral, and disagree). The responses that obtained the maximum number of “disagree” were eliminated from the questionnaire. The ones that obtained the maximum number of “neutral” were further discussed with the study investigators and the questions were reframed or eliminated based on the suggestions provided.

### Statistical analyses

Descriptive statistics were used to analyze the demographics, PA promotion, practices, and beliefs of the nurses. The descriptive data were presented as mean and standard deviation or counts and frequencies for the continuous and categorical data, respectively. Subgroup comparisons were based on the qualification of the nursing staff working in the tertiary care hospitals *i.e.*, either Bachelor of Science in Nursing (BSc Nursing) or General Nursing and Midwifery (GNM). Years of practice, hospital type, and location of the hospital were not chosen for subgroup comparisons as the sample size in these subgroups was unequal. Chi-square test of association for independent samples was performed for subgroup comparisons for all categorical variables. The Mann–Whitney U test was conducted for all continuous variables (non-normally distributed data). Data was analyzed using the software *Jamovi* version 1.6.23, with *p* < 0.05 considered statistically significant.

## Results

A total of 400 nurses working in a tertiary care hospital were approached to participate in the survey, of which 388 responses were obtained. This accounts to a response rate of 97%.

Details regarding the demographic characteristics of the nurses are presented in Table 1. Most of the respondents were female (97.7%). The mean age of the nursing staff was 34.4 ± 9.5 years, and the mean number of years of nursing practice was 11.3 ± 8.7 years, with 2.0 ± 3.9 years of specific practice with cancer patients. Most of the nurses were regularly physically active, either performing the American College of Sports Medicine (ACSM) recommended duration of moderate intensity (78%) or high-intensity exercise per week (12.6%).

**Table 1:** Demographic characteristics of the nurses.

	BSc		GNM	
Characteristic	**Frequency (n)**	**Percentage (%)**	**Frequency (n)**	**Percentage** **(%)**
Age (years)				
(*n* = 384) (Missing = 4)				
Younger than 25	49	12.8%	31	8.0%
26–35	74	19.3%	90	23.4%
36–45	34	8.9%	41	10.7%
46–55	18	4.7%	37	9.6%
56–65	3	0.8%	4	1.0%
Gender				
(*n* = 384) (Missing=4)				
Female	175	45.6%	200	52.0%
Male	4	1.0%	5	1.3%
Highest qualification (*n* = 384) (Missing = 4)	179	46.6%	205	53.4%
(BSc and GNM)				
Years of practice				
(*n* = 377) (Missing = 11)				
Fewer than 5	67	17.8%	34	9.0%
5–14.9	69	18.3%	100	26.5%
15–24.9	22	5.8%	30	7.9%
More than equal to 25	17	4.5%	35	9.2%
Years of practice in tumor/cancer group				
(*n* = 384) (Missing = 4)				
Fewer than 5	35	9.1%	51	13.2%
5–14.9	19	4.9%	20	5.2%
15–24.9	3	0.7%	4	1.0%
More than 25	0	0	2	0.5%
No experience	122	31.7%	128	33.3%
Working Hospital Type				
(*n* = 380) (Missing = 8)				
Public	1	0.2%	4	1.0%
Private	175	46.0%	197	51.8%
Location (*n* = 382) (Missing = 6)				
Metropolitan	33	8.6%	21	5.5%
Regional	127	33.2%	156	40.8%
Rural	16	4.1%	28	7.3%
Physical activity levels				
(*n* = 381) (Missing = 7)				
Five or more 30-minute sessions of moderate-intensity exercise per week	139	36.5%	158	41.4%
Three or more 20-minute sessions of high-intensity exercise per week	15	3.9%	33	8.6%
Not regularly physically active	20	5.2%	13	3.4%

**Notes.**

BSc Bachelor of Science in Nursing GNM General Nursing and Midwifery

Table 2 describes the current PA beliefs, and practices of the nurses. The nurses’ (BSc and GNM) views on who the primary person responsible for promoting PA to their cancer survivors varied. While oncologists were the most common response (47%), physiotherapists (28.9%) and nurses (20.3%) were also reported as being the primary person responsible for promoting PA to cancer survivors. PA was promoted at multiple time points of cancer treatment, with post-treatment (31.7%) being the most common time point among both BSc and GNM nursing staff.

**Table 2:** Nurses’ current physical activity beliefs and practices.

Variable *n* = 374(%) (Missing data/no response = 4)	Educational qualification		Total *n* = 370
	**BSc** *n* = 170 (45.9%)	**GNM** *n* = 200 (54.1%)	
**(A) In your opinion, who is the primary person responsible for promoting physical activity to your patients with cancer?**			
Me	44 (11.9)	31 (8.4)	75 (20.3)
Physiotherapist	50 (13.5)	57 (15.4)	107 (28.9)
Oncologist	**69 (18.6)**	**105 (28.4)**	**174 (47.0)**
Exercise physiologist	0 (0.0)	3 (0.8)	3 (0.8)
Nutritionist/dietician	6 (1.6)	4 (1.1)	10 (2.7)
Don’t know	1 (0.3)	0 (0.0)	1 (0.3)
**(B) Indicate the stage(s) of cancer treatment at which physical activity is promoted to your patients with cancer.**			
**Variable***n* = 385(%) **(Missing data/no response = 3**)	**Educational qualification**		**Total** *n* = 382
	**BSc** *n* = 177(46.3%)	**GNM** *n* = 205(53.7%)	
Pre-treatment	37 (9.7)	48 (12.6)	85 (22.3)
During-treatment	40 (10.5)	43 (11.3)	83 (21.7)
Post-treatment	**49 (12.8)**	**72 (18.6)**	**121 (31.7)**
Pre & Post-treatment	17 (4.5)	16 (4.2)	33 (8.6)
Pre & during-treatment	2 (0.5)	0 (0.0)	2 (0.5)
During & Post-treatment	10 (2.6)	6 (1.6)	16 (4.2)
Every stage	22 (5.8)	20 (5.2)	42 (11.0)

**Notes.**

Group differences are based on Chi-Square test of association for independent samples.

BSc (Bachelor of Science in Nursing) GNM (General Nursing and Midwifery)

Percentages are calculated based on observed % of total value.

(A) *p* value = 0.032.

(B) *p* value = 0.319.

Subgroup comparisons were also performed to determine whether there were any statistical differences in the PA beliefs and practices based on the educational qualification of the nurses. The nurses who had completed their general nursing and midwifery (52.5%) were significantly more likely to consider the oncologists as the primary person responsible for promoting PA than BSc nurses (40.5%) (*p* = 0.032). No significant difference (*p* = 0.319) was observed between the two groups (BSc and GNM), for the stages at which PA was promoted to cancer survivors.

The nurses’ beliefs about the benefits of PA for cancer survivors are summarized in Table 3. Specifically, they agreed or strongly agreed that the major benefits of PA were improved mental health (87.7%), health-related quality of life (87.1%), weight management (85.6%) and ability to perform activities of daily living (85.3%). It was also observed a vast majority of nurses agreed or strongly agreed that they should be promoting PA to their patients (78.9%) and that there is a strong evidence base encouraging such promotion (77.1%).

**Table 3:** Nurses’ beliefs about the benefits of physical activity for cancer survivors.

Beliefs	Strongly disagree *n*(%)	Disagree *n*(%)	Agree *n*(%)	Strongly agree *n*(%)
Improves health-related quality of life (*n* = 385)	37 (9.6)	13 (3.4)	257 (66.8)	78 (20.3)
Improves weight management (*n* = 384)	22 (5.7)	33 (8.6)	267 (69.5)	62 (16.1)
Improves fatigue level (*n* = 383)	32 (8.4)	43 (11.2)	235 (61.4)	73 (19.1)
Improves mental health (*n* = 382)	27 (7.1)	20 (5.2)	239 (62.6)	96 (25.1)
Improves activities of daily living (*n* = 384)	28 (7.3)	28 (7.3)	259 (67.3)	69 (18.0)
Reduces risk of cancer recurrence (*n* = 380)	27 (7.1)	71 (18.7)	221 (58.2)	61 (16.1)
Reduces risk of other chronic diseases (*n* = 380)	26 (6.8)	58 (15.3)	221 (58.2)	75 (19.7)
Reduces tumor specific comorbidities (*n* = 341)	34 (10.0)	76 (22.3)	188 (55.1)	43 (12.6)
No benefits (*n* = 299)	112 (37.4)	81 (27.1)	94 (31.4)	12 (4.0)
My patients with cancer are generally uninterested in physical activity (*n* = 381)	39 (10.2)	124 (32.5)	203 (53.3)	15 (3.9)
Whether or not I promote physical activity to my patients with cancer is entirely up to me (*n* = 376)	59 (15.7)	78 (20.7)	220 (58.5)	19 (5.1)
My fellow nurses believe I should be promoting physical activity to my patients with cancer (*n* = 379)	26 (6.9)	54 (14.2)	260 (68.6)	39 (10.3)
There is a strong evidence base suggesting that I should promote physical activity to my patients with cancer (*n* = 377)	36 (9.5)	50 (14.3)	249 (66.0)	42 (11.1)

Table 4 presents a comparison of the two subgroups of nurses’ beliefs about the benefits of PA for cancer survivors. There were no significant differences between the groups with respect to the nurses’ beliefs about the benefits of PA for their patients. The only exception was found for the question “There are no benefits of exercise”, in which the GNM nurses were more likely than BSc nurses to agree with the statement (*p* = 0.02).

**Table 4:** Comparison of the Nurses’ beliefs about the benefits of physical activity for cancer survivors based on their educational qualifications.

Belief	Educational qualification				p
	**BSc**		**GNM**		
	$\bar {X}$	**SD**	$\bar {X}$	**SD**	
Improves health-related quality of life	2.97	0.85	2.98	0.73	0.62
Improves weight management	2.94	0.81	2.98	0.57	0.83
Improves fatigue level	2.88	0.93	2.94	0.65	0.89
Improves mental health	3.13	0.87	3.00	0.67	0.006
Improves activities of daily living	2.99	0.84	2.93	0.64	0.07
Reduces risk of cancer recurrence	2.77	0.89	2.89	0.67	0.16
Reduces risk of other chronic diseases	2.89	0.88	2.92	0.70	0.98
Reduces tumor specific comorbidities	2.70	0.85	2.72	0.78	0.61
No benefits	1.92	0.95	2.14	0.88	**0.02**
My patients with cancer are generally uninterested in physical activity	2.50	0.78	2.51	0.69	0.96
Whether or not I promote physical activity to my patients with cancer is entirely up to me	2.45	0.86	2.59	0.77	0.08
My fellow nurses believe I should be promoting physical activity to my patients with cancer	2.79	0.74	2.85	0.67	0.49
There is a strong evidence base suggesting that I should promote physical activity to my patients with cancer	2.71	0.84	2.85	0.69	012

**Notes.**

Group differences were calculated using the Mann–Whitney *U* test.

All items are rated on a 4 -point Likert-type scale from 1(strongly disagree) to 4(strongly agree).

BSc (Bachelor of Science in Nursing) GNM (General Nursing and Midwifery)

Table 5 summarizes the most frequently cited barriers by nurses in promoting PA to cancer survivors. Most of the nurses (BSc and GNM) were neutral regarding the potential barriers listed for this question. The most frequently cited barriers for the entire group were lack of knowledge (42.2%), lack of time (41.6%) and risk to survivors (40.3%). Sub-group comparisons typically revealed no differences between the BSc and GNM nurses. The only exception to this was how a greater percentage of GNM qualified nurses (38.9%) perceived the risk to the survivors as a barrier preventing them from promoting PA to their cancer survivors on a regular basis as compared to the BSc qualified nurses (35.8%).

**Table 5:** Most frequently cited barriers by nurses in promoting physical activity among cancer survivors.

Barrier	Professional qualification						*p*	*χ* ^2^
	BSc			GNM				
	Most likely *n*(%)	Neutral *n*(%)	Least likely *n*(%)	Most likely *n*(%)	Neutral *n*(%)	Least likely *n*(%)		
Lack of time (*n* = 356)	70 (41.4)	82 (48.5)	17 (10.0)	78 (41.7)	88 (47.1)	21 (11.2)	0.925	0.156
Risk to patient (*n* = 355)	58 (35.8)	73 (45.1)	31 (19.1)	75 (38.9)	104 (53.9)	14 (7.2)	**0.003**	11.4
Lack of adequate support structure (*n* = 362)	54 (32.1)	95 (56.5)	19 (11.3)	67 (34.5)	112 (57.7)	15 (7.7)	0.496	1.40
Lack of Knowledge (*n* = 332)	62 (40.2)	64 (41.5)	28 (18.1)	78 (43.8)	82 (46.1)	18 (10.1)	0.105	4.51
Lack of expertise (*n* = 321)	47 (30.7)	82 (53.6)	24 (15.7)	54 (32.1)	94 (55.9)	20 (11.9)	0.616	0.968
Do not promote physical activity (*n* = 295)	36 (27.7)	59 (45.4)	35 (26.9)	56 (33.9)	80 (48.5)	29 (17.6)	0.136	3.99
Not my job (*n* = 287)	31 (23.5)	59 (44.7)	42 (31.8)	42 (27.1)	74 (47.7)	39 (25.2)	0.443	1.63
Do not have barriers in promoting physical activity (*n* = 285)	34 (26.6)	65 (50.8)	29 (22.6)	45 (28.7)	79 (50.3)	33 (21.0)	0.904	0.202
Other (*n* = 198)	30 (34.8)	44 (51.2)	12 (13.9)	34 (30.3)	67 (59.8)	11 (9.8)	0.433	1.67

**Notes.**

Group differences are based on Chi-Square test of association for independent samples.

The respondents were asked to indicate the three most likely factors that prevent them from promoting physical activity (with 1 being the most likely and 3 being the least likely).

BSc (Bachelor of Science in Nursing) GNM (General Nursing and Midwifery)

## Discussion

This study was a cross sectional survey conducted among nursing staff in a tertiary care hospital in Mangalore, Karnataka, India. Our study aimed to better understand the beliefs of the Indian Nurses regarding PA promotion practices among cancer survivors. Most of the nurses working in the tertiary care hospital were females, between 25–35 years (BSc: 19.3% and GNM 23.4%) and had more than five years of nursing experience. The hospital had a similar percentage of GNM (53.4%) and BSc (46.6%) nurses.

The results of our study demonstrate that the nursing staff (especially those who completed the GNM qualification) believed that oncologists were primarily responsible for the promotion of PA to cancer survivors, with physiotherapists and nurses also quite important. These results are somewhat consistent with previous studies wherein the nurses may play an important role in providing essential information to cancer survivors regarding PA (Karvinen et al., 2012; Keogh et al., 2017b). However, Australasian nurses for whom this questionnaire was first used, felt that they (nurses) were the primary professional group responsible promoting PA to their cancer survivors (Keogh et al., 2017a). Such a result suggests that PA programs targeting improved cancer survivorship outcomes in India may also need to better engage oncologists who can then better collaborate with their allied health teams (*e.g.*, physiotherapists and nurses).

There was no single treatment stage in which the nurses typically promoted PA to cancer survivors, with the majority of nurses only promoting PA at one of the three treatment phases. Specifically, 31.7% of the nurses promoted PA during the post-treatment phase, followed by 22.2% pre-treatment and 21.7% during-treatment. Current research evidence supports the promotion of PA during various stages of treatment because in each stage of treatment, regular PA may offset some of the different physical and psychosocial challenges and symptoms associated with each stage (Stevinson & Fox, 2005; Thorsen et al., 2008; Karvinen et al., 2012; Karvinen et al., 2017). This suggests that Indian nurses may require more education in the importance of regular PA across all stages of cancer treatment/survivorship and/or more assistance in promoting PA across the different phases.

The relatively strong PA promotion from the nurses (albeit typically only at one stage of cancer treatment) appears to be consistent with their own beliefs whereby, a majority of the nurses “strongly agreed” or “agreed” that there is a strong evidence base for the promotion of PA among cancer survivors. In addition, a majority of the nursing staff strongly agreed or agreed that they should be promoting PA to their patients regularly. However, they also believed that the majority (57.2%) of their patients were generally “uninterested” in PA. This perception might be due to the lack of incorporation of PA in most of the health care facilities across India for the treatment of cancer survivors. These results suggest that Indian hospitals may still need to look at better supporting PA referral pathways and the promotion of PA by a variety of healthcare staff to their cancer patients and that their staff (including nurses) may need to be more formally trained in how to promote and encourage the performance of regular PA during the various stages of cancer treatment.

The nurses believed that the strongest benefits achieved by the regular performance of PA for cancer survivors were improvements in mental health, health-related quality of life, weight management, and ability to perform activities of daily living. However, 35.4% of the nursing staff felt that the performance of PA had “no benefits” for cancer survivors. This finding may reflect some of the findings regarding the barriers that nurses faced in promoting PA to their patients (Keogh et al., 2017a). Most of the nurses were neutral regarding the barriers they faced while promoting PA to the cancer survivors. However, of the barriers that the nurses described as “most likely”, a lack of knowledge regarding PA and its benefits, a lack of time and potential patient risks were most commonly described. These results were consistent with a previous study whereby, majority of the oncology nurses were unaware regarding the specific benefits of PA for cancer survivors (Stevinson & Fox, 2005). The lack of time is a commonly reported barrier by many health professionals, including nurses (Puhringer et al., 2015). It was interesting to observe that the “risk to the patient” was perceived by the GNM nurses as more of a barrier preventing them from promoting PA to cancer survivors than felt by the BSc nurses. These results further indicate Indian hospitals may need to focus their PA education programs on GNM rather than BSc trained nurses, perhaps due to the GNM nurses having one less year of tertiary training than the BSc nurses.

## Conclusion

The results of the current study may add to the limited evidence available regarding the promotion of PA for cancer survivors by nurses, especially in developing countries such as India. The major result of this study suggests that while the nurses were somewhat interested in promoting PA for cancer survivors, the GNM nurses as compared to the BSc nurses, felt that the oncologists followed by the physiotherapists played a more important role in promoting PA to cancer survivors than nurses. The stage at which the nursing staff promoted PA did show considerable variation based on their educational qualification. The GNM nurses, as compared to the BSc nurses, felt that the post treatment stage followed by pre- and during treatment were ideal stages for PA promotion to cancer survivors. Moreover, in terms of barriers, GNM nurses as compared to the BSc nurses felt that risk to the patient might be a major barrier for the promotion of PA to cancer survivors. Nurses also believed that additional educational and organizational support may be useful to further enhance the interdisciplinary promotion of PA to cancer survivors by professional groups including nurses, oncologists, and other health professionals.

## Supplemental Information

10.7717/peerj.13348/supp-1 Supplemental Information 1 Raw Data

10.7717/peerj.13348/supp-2 Supplemental Information 2 Statistical Analysis

10.7717/peerj.13348/supp-3 Supplemental Information 3 Survey Questionaare

10.7717/peerj.13348/supp-4 Supplemental Information 4 Mann Whitney Test

## Additional Information and Declarations

### Data Availability

The following information was supplied regarding data availability: The raw data and the analysis is available in the Supplementary File.
