# Supplementary material for: Indian nurses’ beliefs on physical activity promotion practices for cancer survivors in a tertiary care hospital—a cross-sectional survey
Source: PeerJ. 2022 May 23;10:e13348. doi: 10.7717/peerj.13348 (PMC9135035; doi:10.7717/peerj.13348)
Supplement: Supplemental Information 2 [file peerj-10-13348-s002.docx]

**Descriptives - Demographics**

Q 1,2,3,4,5,6,7

| Descriptives | | | | | | | | | | | | | | | |
| --- | --- | --- | --- | --- | --- | --- | --- | --- | --- | --- | --- | --- | --- | --- | --- |
|  |  |  |  |  |  |  |  |  |  |  |  |  |  |  |  |
|  | | **AGE-ANALYSIS** | | **GENDER** | | **Q3** | | **YOP** | | **Q5 Cat** | | **Q6** | | **LOCATION** | |
| N |  | 384 |  | 388 |  | 384 |  | 377 |  | 388 |  | 380 |  | 382 |  |
| Missing |  | 4 |  | 0 |  | 4 |  | 11 |  | 0 |  | 8 |  | 6 |  |
| Shapiro-Wilk W |  | . |  | . |  | . |  | . |  | . |  | . |  | . |  |
| Shapiro-Wilk p |  |  |  |  |  |  |  |  |  |  |  |  |  |  |  |
|  | | | | | | | | | | | | | | | |

**Frequencies**

| Frequencies of AGE-ANALYSIS | | | | | | | |
| --- | --- | --- | --- | --- | --- | --- | --- |
|  |  |  |  |  |  |  |  |
| **Levels** | | **Counts** | | **% of Total** | | **Cumulative %** | |
| LESSTHAN25 |  | 80 |  | 20.8 % |  | 20.8 % |  |
| LESSTHAN35 |  | 166 |  | 43.2 % |  | 64.1 % |  |
| LESSTHAN45 |  | 76 |  | 19.8 % |  | 83.9 % |  |
| LESSTHAN55 |  | 55 |  | 14.3 % |  | 98.2 % |  |
| LESSTHAN65 |  | 7 |  | 1.8 % |  | 100.0 % |  |
|  | | | | | | | |

| Frequencies of GENDER | | | | | | | |
| --- | --- | --- | --- | --- | --- | --- | --- |
|  |  |  |  |  |  |  |  |
| **Levels** | | **Counts** | | **% of Total** | | **Cumulative %** | |
| F |  | 379 |  | 97.7 % |  | 97.7 % |  |
| M |  | 9 |  | 2.3 % |  | 100.0 % |  |
|  | | | | | | | |

| Frequencies of Q3 | | | | | | | |
| --- | --- | --- | --- | --- | --- | --- | --- |
|  |  |  |  |  |  |  |  |
| **Levels** | | **Counts** | | **% of Total** | | **Cumulative %** | |
| BSC NURSING |  | 179 |  | 46.6 % |  | 46.6 % |  |
| GNM |  | 205 |  | 53.4 % |  | 100.0 % |  |
|  | | | | | | | |

| Frequencies of YOP | | | | | | | |
| --- | --- | --- | --- | --- | --- | --- | --- |
|  |  |  |  |  |  |  |  |
| **Levels** | | **Counts** | | **% of Total** | | **Cumulative %** | |
| LESSTHAN5 |  | 102 |  | 27.1 % |  | 27.1 % |  |
| LESSTHAN14 |  | 170 |  | 45.1 % |  | 72.1 % |  |
| LESSTHAN24 |  | 53 |  | 14.1 % |  | 86.2 % |  |
| MORETHAN25 |  | 52 |  | 13.8 % |  | 100.0 % |  |
|  | | | | | | | |

| Frequencies of Q5 Cat | | | | | | | |
| --- | --- | --- | --- | --- | --- | --- | --- |
|  |  |  |  |  |  |  |  |
| **Levels** | | **Counts** | | **% of Total** | | **Cumulative %** | |
| 1 |  | 21 |  | 5.4 % |  | 5.4 % |  |
| 10 |  | 17 |  | 4.4 % |  | 9.8 % |  |
| 11 |  | 1 |  | 0.3 % |  | 10.1 % |  |
| 12 |  | 1 |  | 0.3 % |  | 10.3 % |  |
| 13 |  | 1 |  | 0.3 % |  | 10.6 % |  |
| 14 |  | 1 |  | 0.3 % |  | 10.8 % |  |
| 15 |  | 3 |  | 0.8 % |  | 11.6 % |  |
| 16 |  | 1 |  | 0.3 % |  | 11.9 % |  |
| 2 |  | 19 |  | 4.9 % |  | 16.8 % |  |
| 20 |  | 3 |  | 0.8 % |  | 17.5 % |  |
| 25 |  | 2 |  | 0.5 % |  | 18.0 % |  |
| 3 |  | 18 |  | 4.6 % |  | 22.7 % |  |
| 4 |  | 13 |  | 3.4 % |  | 26.0 % |  |
| 5 |  | 17 |  | 4.4 % |  | 30.4 % |  |
| 6 |  | 12 |  | 3.1 % |  | 33.5 % |  |
| 7 |  | 2 |  | 0.5 % |  | 34.0 % |  |
| 8 |  | 4 |  | 1.0 % |  | 35.1 % |  |
| NIL |  | 252 |  | 64.9 % |  | 100.0 % |  |
|  | | | | | | | |

| Frequencies of Q6 | | | | | | | |
| --- | --- | --- | --- | --- | --- | --- | --- |
|  |  |  |  |  |  |  |  |
| **Levels** | | **Counts** | | **% of Total** | | **Cumulative %** | |
| PRIVATE |  | 375 |  | 98.7 % |  | 98.7 % |  |
| PUBLIC |  | 5 |  | 1.3 % |  | 100.0 % |  |
|  | | | | | | | |

| Frequencies of LOCATION | | | | | | | |
| --- | --- | --- | --- | --- | --- | --- | --- |
|  |  |  |  |  |  |  |  |
| **Levels** | | **Counts** | | **% of Total** | | **Cumulative %** | |
| METRO |  | 55 |  | 14.4 % |  | 14.4 % |  |
| REGIONAL |  | 283 |  | 74.1 % |  | 88.5 % |  |
| RURAL |  | 44 |  | 11.5 % |  | 100.0 % |  |
|  |  |  |  |  |  |  |  |
|  | | | | | | | |
| Descriptives – Q8 and Q9 Q8-primary person promoting PA & Q9-stage at which PA is promoted   \| Descriptives \| \| \| \| \| \| \| --- \| --- \| --- \| --- \| --- \| --- \| \|  \|  \|  \|  \|  \|  \| \|  \| \| **PHR 8-PRIMARY PERSON PROMOTING PA** \| \| **PHR 9** \| \| \| N \|  \| 374 \|  \| 385 \|  \| \| Missing \|  \| 14 \|  \| 3 \|  \| \|  \| \| \| \| \| \|    Frequencies  \| Frequencies of PHR 8-PRIMARY PERSON PROMOTING PA \| \| \| \| \| \| \| \| \| --- \| --- \| --- \| --- \| --- \| --- \| --- \| --- \| \|  \|  \|  \|  \|  \|  \|  \|  \| \| **Levels** \| \| **Counts** \| \| **% of Total** \| \| **Cumulative %** \| \| \| NURSE \|  \| 76 \|  \| 20.3 % \|  \| 20.3 % \|  \| \| PT \|  \| 108 \|  \| 28.9 % \|  \| 49.2 % \|  \| \| ONCOLOGIST \|  \| 176 \|  \| 47.1 % \|  \| 96.3 % \|  \| \| EXE PHY \|  \| 3 \|  \| 0.8 % \|  \| 97.1 % \|  \| \| NUTRITIONIST \|  \| 10 \|  \| 2.7 % \|  \| 99.7 % \|  \| \| DONT KNOW \|  \| 1 \|  \| 0.3 % \|  \| 100.0 % \|  \| \|  \| \| \| \| \| \| \| \|      \| Frequencies of PHR 9 \| \| \| \| \| \| \| \| \| --- \| --- \| --- \| --- \| --- \| --- \| --- \| --- \| \|  \|  \|  \|  \|  \|  \|  \|  \| \| **Levels** \| \| **Counts** \| \| **% of Total** \| \| **Cumulative %** \| \| \| Pre Rx \|  \| 85 \|  \| 22.1 % \|  \| 22.1 % \|  \| \| Pre/Post Rx \|  \| 34 \|  \| 8.8 % \|  \| 30.9 % \|  \| \| Every Stage \|  \| 44 \|  \| 11.4 % \|  \| 42.3 % \|  \| \| Pre/During Rx \|  \| 2 \|  \| 0.5 % \|  \| 42.9 % \|  \| \| Post Rx \|  \| 121 \|  \| 31.4 % \|  \| 74.3 % \|  \| \| During/Post Rx \|  \| 16 \|  \| 4.2 % \|  \| 78.4 % \|  \| \| During \|  \| 83 \|  \| 21.6 % \|  \| 100.0 % \|  \| \|  \| \| \| \| \| \| \| \| \|  \| \| \| \| \| \| \| \| | | | | | | | |

# Chi square - primary person promoting PA (Q8)

| Contingency Tables | | | | | | | | | |
| --- | --- | --- | --- | --- | --- | --- | --- | --- | --- |
|  | | | | **Q3** | | | |  | |
| **PHR 8-PRIMARY PERSON PROMOTING PA** | |  | | **BSC NURSING** | | **GNM** | | **Total** | |
| NURSE |  | Observed |  | 44 |  | 31 |  | 75 |  |
|  | | % of total |  | 11.9 % |  | 8.4 % |  | 20.3 % |  |
| PT |  | Observed |  | 50 |  | 57 |  | 107 |  |
|  | | % of total |  | 13.5 % |  | 15.4 % |  | 28.9 % |  |
| ONCOLOGIST |  | Observed |  | 69 |  | 105 |  | 174 |  |
|  | | % of total |  | 18.6 % |  | 28.4 % |  | 47.0 % |  |
| EXE PHY |  | Observed |  | 0 |  | 3 |  | 3 |  |
|  | | % of total |  | 0.0 % |  | 0.8 % |  | 0.8 % |  |
| NUTRITIONIST |  | Observed |  | 6 |  | 4 |  | 10 |  |
|  | | % of total |  | 1.6 % |  | 1.1 % |  | 2.7 % |  |
| DONT KNOW |  | Observed |  | 1 |  | 0 |  | 1 |  |
|  | | % of total |  | 0.3 % |  | 0.0 % |  | 0.3 % |  |
| Total |  | Observed |  | 170 |  | 200 |  | 370 |  |
|  | | % of total |  | 45.9 % |  | 54.1 % |  | 100.0 % |  |
|  | | | | | | | | | |

| χ² Tests | | | | | | | |
| --- | --- | --- | --- | --- | --- | --- | --- |
|  |  |  |  |  |  |  |  |
|  | | **Value** | | **df** | | **p** | |
| χ² |  | 12.2 |  | 5 |  | 0.032 |  |
| N |  | 370 |  |  | |  | |
|  | | | | | | | |

**Chi square- (Q9) indicate the stage at which PA is promoted**

| Contingency Tables | | | | | | | | | |
| --- | --- | --- | --- | --- | --- | --- | --- | --- | --- |
|  | | | | **Q3** | | | |  | |
| **PHR 9** | |  | | **BSC NURSING** | | **GNM** | | **Total** | |
| Pre Rx |  | Observed |  | 37 |  | 48 |  | 85 |  |
|  | | % of total |  | 9.7 % |  | 12.6 % |  | 22.3 % |  |
| Pre/Post Rx |  | Observed |  | 17 |  | 16 |  | 33 |  |
|  | | % of total |  | 4.5 % |  | 4.2 % |  | 8.6 % |  |
| Every Stage |  | Observed |  | 22 |  | 20 |  | 42 |  |
|  | | % of total |  | 5.8 % |  | 5.2 % |  | 11.0 % |  |
| Pre/During Rx |  | Observed |  | 2 |  | 0 |  | 2 |  |
|  | | % of total |  | 0.5 % |  | 0.0 % |  | 0.5 % |  |
| Post Rx |  | Observed |  | 49 |  | 72 |  | 121 |  |
|  | | % of total |  | 12.8 % |  | 18.8 % |  | 31.7 % |  |
| During/Post Rx |  | Observed |  | 10 |  | 6 |  | 16 |  |
|  | | % of total |  | 2.6 % |  | 1.6 % |  | 4.2 % |  |
| During |  | Observed |  | 40 |  | 43 |  | 83 |  |
|  | | % of total |  | 10.5 % |  | 11.3 % |  | 21.7 % |  |
| Total |  | Observed |  | 177 |  | 205 |  | 382 |  |
|  | | % of total |  | 46.3 % |  | 53.7 % |  | 100.0 % |  |
|  | | | | | | | | | |

| χ² Tests | | | | | | | |
| --- | --- | --- | --- | --- | --- | --- | --- |
|  |  |  |  |  |  |  |  |
|  | | **Value** | | **df** | | **p** | |
| χ² |  | 7.01 |  | 6 |  | 0.319 |  |
| N |  | 382 |  |  | |  | |
|  | | | | | | | |

**Descriptives - Q12,Q13- regular PA**

REGULARLY PA AND EAT HEALTHY REGULARLY - Q12 AND 13

| Descriptives | | | | | |
| --- | --- | --- | --- | --- | --- |
|  |  |  |  |  |  |
|  | | **REGULARLY PA** | | **EAT HEALTHY** | |
| N |  | 381 |  | 382 |  |
| Missing |  | 7 |  | 6 |  |
|  | | | | | |

**Frequencies**

| Frequencies of REGULARLY PA | | | | | | | |
| --- | --- | --- | --- | --- | --- | --- | --- |
|  |  |  |  |  |  |  |  |
| **Levels** | | **Counts** | | **% of Total** | | **Cumulative %** | |
| 5-TO-30MIN |  | 297 |  | 78.0 % |  | 78.0 % |  |
| 3/moreMINS |  | 48 |  | 12.6 % |  | 90.6 % |  |
| NO PA |  | 36 |  | 9.4 % |  | 100.0 % |  |
|  | | | | | | | |

**One-Way ANOVA (Non-parametric) - Q16**

Q16 - what benefits may PA have for your patients with cancer

| Kruskal-Wallis | | | | | | | | | |
| --- | --- | --- | --- | --- | --- | --- | --- | --- | --- |
|  |  |  |  |  |  |  |  |  |  |
|  | | **χ²** | | **df** | | **p** | | **ε²** | |
| 16 HRQL |  | 0.2462 |  | 1 |  | 0.620 |  | 6.48e-4 |  |
| 16 WT |  | 0.0443 |  | 1 |  | 0.833 |  | 1.17e-4 |  |
| 16 FATIGUE |  | 0.0180 |  | 1 |  | 0.893 |  | 4.76e-5 |  |
| 16 MENTAL |  | 7.5763 |  | 1 |  | 0.006 |  | 0.02010 |  |
| 16 ADL |  | 3.0870 |  | 1 |  | 0.079 |  | 0.00815 |  |
| 16 CA |  | 1.9039 |  | 1 |  | 0.168 |  | 0.00508 |  |
| 16 CHRONIC |  | 6.02e-4 |  | 1 |  | 0.980 |  | 1.61e-6 |  |
| 16 TUMOR |  | 0.2579 |  | 1 |  | 0.612 |  | 7.68e-4 |  |
| 16 NO B |  | 4.8743 |  | 1 |  | 0.027 |  | 0.01698 |  |
|  | | | | | | | | | |

# Chi square - (Q18) BARRIERS IN PROMOTING PA

CHI SQUARE (Q18) - LACK OF TIME

| Contingency Tables | | | | | | | | | |
| --- | --- | --- | --- | --- | --- | --- | --- | --- | --- |
|  | | | | **Q3** | | | |  | |
| **18 MOT TIME** | |  | | **BSC NURSING** | | **GNM** | | **Total** | |
| most likely |  | Observed |  | 70 |  | 78 |  | 148 |  |
|  | | % of total |  | 19.7 % |  | 21.9 % |  | 41.6 % |  |
| neutral |  | Observed |  | 82 |  | 88 |  | 170 |  |
|  | | % of total |  | 23.0 % |  | 24.7 % |  | 47.8 % |  |
| least likely |  | Observed |  | 17 |  | 21 |  | 38 |  |
|  | | % of total |  | 4.8 % |  | 5.9 % |  | 10.7 % |  |
| Total |  | Observed |  | 169 |  | 187 |  | 356 |  |
|  | | % of total |  | 47.5 % |  | 52.5 % |  | 100.0 % |  |
|  | | | | | | | | | |

| χ² Tests | | | | | | | |
| --- | --- | --- | --- | --- | --- | --- | --- |
|  |  |  |  |  |  |  |  |
|  | | **Value** | | **df** | | **p** | |
| χ² |  | 0.156 |  | 2 |  | 0.925 |  |
| N |  | 356 |  |  | |  | |
|  | | | | | | | |

**Chi square (Q18)-RISK TO PATIENT**

| Contingency Tables | | | | | | | | | |
| --- | --- | --- | --- | --- | --- | --- | --- | --- | --- |
|  | | | | **Q3** | | | |  | |
| **MOT 18 RISK TO PATIENT** | |  | | **BSC NURSING** | | **GNM** | | **Total** | |
| most likely |  | Observed |  | 58 |  | 75 |  | 133 |  |
|  | | % of total |  | 16.3 % |  | 21.1 % |  | 37.5 % |  |
| neutral |  | Observed |  | 73 |  | 104 |  | 177 |  |
|  | | % of total |  | 20.6 % |  | 29.3 % |  | 49.9 % |  |
| least likely |  | Observed |  | 31 |  | 14 |  | 45 |  |
|  | | % of total |  | 8.7 % |  | 3.9 % |  | 12.7 % |  |
| Total |  | Observed |  | 162 |  | 193 |  | 355 |  |
|  | | % of total |  | 45.6 % |  | 54.4 % |  | 100.0 % |  |
|  | | | | | | | | | |

| χ² Tests | | | | | | | |
| --- | --- | --- | --- | --- | --- | --- | --- |
|  |  |  |  |  |  |  |  |
|  | | **Value** | | **df** | | **p** | |
| χ² |  | 11.4 |  | 2 |  | 0.003 |  |
| N |  | 355 |  |  | |  | |
|  | | | | | | | |

**Chi square (Q18) - ADEQUATE SS**

| Contingency Tables | | | | | | | | | |
| --- | --- | --- | --- | --- | --- | --- | --- | --- | --- |
|  | | | | **Q3** | | | |  | |
| **MOT 18 ADEQUATE SS** | |  | | **BSC NURSING** | | **GNM** | | **Total** | |
| most likely |  | Observed |  | 54 |  | 67 |  | 121 |  |
|  | | % of total |  | 14.9 % |  | 18.5 % |  | 33.4 % |  |
| neutral |  | Observed |  | 95 |  | 112 |  | 207 |  |
|  | | % of total |  | 26.2 % |  | 30.9 % |  | 57.2 % |  |
| least likely |  | Observed |  | 19 |  | 15 |  | 34 |  |
|  | | % of total |  | 5.2 % |  | 4.1 % |  | 9.4 % |  |
| Total |  | Observed |  | 168 |  | 194 |  | 362 |  |
|  | | % of total |  | 46.4 % |  | 53.6 % |  | 100.0 % |  |
|  | | | | | | | | | |

| χ² Tests | | | | | | | |
| --- | --- | --- | --- | --- | --- | --- | --- |
|  |  |  |  |  |  |  |  |
|  | | **Value** | | **df** | | **p** | |
| χ² |  | 1.40 |  | 2 |  | 0.496 |  |
| N |  | 362 |  |  | |  | |
|  | | | | | | | |

**Chi square - q18 LACK OF KNOWLEDGE**

| Contingency Tables | | | | | | | | | |
| --- | --- | --- | --- | --- | --- | --- | --- | --- | --- |
|  | | | | **Q3** | | | |  | |
| **MOT 18 KNOWLEDGE** | |  | | **BSC NURSING** | | **GNM** | | **Total** | |
| most likely |  | Observed |  | 62 |  | 78 |  | 140 |  |
|  | | % of total |  | 18.7 % |  | 23.5 % |  | 42.2 % |  |
| neutral |  | Observed |  | 64 |  | 82 |  | 146 |  |
|  | | % of total |  | 19.3 % |  | 24.7 % |  | 44.0 % |  |
| least likely |  | Observed |  | 28 |  | 18 |  | 46 |  |
|  | | % of total |  | 8.4 % |  | 5.4 % |  | 13.9 % |  |
| Total |  | Observed |  | 154 |  | 178 |  | 332 |  |
|  | | % of total |  | 46.4 % |  | 53.6 % |  | 100.0 % |  |
|  | | | | | | | | | |

| χ² Tests | | | | | | | |
| --- | --- | --- | --- | --- | --- | --- | --- |
|  |  |  |  |  |  |  |  |
|  | | **Value** | | **df** | | **p** | |
| χ² |  | 4.51 |  | 2 |  | 0.105 |  |
| N |  | 332 |  |  | |  | |
|  | | | | | | | |

**Chi square - Q18 LACK OF EXPERTISE**

| Contingency Tables | | | | | | | | | |
| --- | --- | --- | --- | --- | --- | --- | --- | --- | --- |
|  | | | | **Q3** | | | |  | |
| **MOT 18 EXPERTISE** | |  | | **BSC NURSING** | | **GNM** | | **Total** | |
| most likely |  | Observed |  | 47 |  | 54 |  | 101 |  |
|  | | % of total |  | 14.6 % |  | 16.8 % |  | 31.5 % |  |
| neutral |  | Observed |  | 82 |  | 94 |  | 176 |  |
|  | | % of total |  | 25.5 % |  | 29.3 % |  | 54.8 % |  |
| least likely |  | Observed |  | 24 |  | 20 |  | 44 |  |
|  | | % of total |  | 7.5 % |  | 6.2 % |  | 13.7 % |  |
| Total |  | Observed |  | 153 |  | 168 |  | 321 |  |
|  | | % of total |  | 47.7 % |  | 52.3 % |  | 100.0 % |  |
|  | | | | | | | | | |

| χ² Tests | | | | | | | |
| --- | --- | --- | --- | --- | --- | --- | --- |
|  |  |  |  |  |  |  |  |
|  | | **Value** | | **df** | | **p** | |
| χ² |  | 0.968 |  | 2 |  | 0.616 |  |
| N |  | 321 |  |  | |  | |
|  | | | | | | | |

**Chi square - (Q18) I DO NOT PROMOTE**

| Contingency Tables | | | | | | | | | |
| --- | --- | --- | --- | --- | --- | --- | --- | --- | --- |
|  | | | | **Q3** | | | |  | |
| **MOT 21 PROMOTE** | |  | | **BSC NURSING** | | **GNM** | | **Total** | |
| most likely |  | Observed |  | 36 |  | 56 |  | 92 |  |
|  | | % of total |  | 12.2 % |  | 19.0 % |  | 31.2 % |  |
| neutral |  | Observed |  | 59 |  | 80 |  | 139 |  |
|  | | % of total |  | 20.0 % |  | 27.1 % |  | 47.1 % |  |
| least likely |  | Observed |  | 35 |  | 29 |  | 64 |  |
|  | | % of total |  | 11.9 % |  | 9.8 % |  | 21.7 % |  |
| Total |  | Observed |  | 130 |  | 165 |  | 295 |  |
|  | | % of total |  | 44.1 % |  | 55.9 % |  | 100.0 % |  |
|  | | | | | | | | | |

| χ² Tests | | | | | | | |
| --- | --- | --- | --- | --- | --- | --- | --- |
|  |  |  |  |  |  |  |  |
|  | | **Value** | | **df** | | **p** | |
| χ² |  | 3.99 |  | 2 |  | 0.136 |  |
| N |  | 295 |  |  | |  | |
|  | | | | | | | |

**Chi square (Q18)-NOT MY JOB**

| Contingency Tables | | | | | | | | | |
| --- | --- | --- | --- | --- | --- | --- | --- | --- | --- |
|  | | | | **Q3** | | | |  | |
| **MOT 21 JOB** | |  | | **BSC NURSING** | | **GNM** | | **Total** | |
| most likely |  | Observed |  | 31 |  | 42 |  | 73 |  |
|  | | % of total |  | 10.8 % |  | 14.6 % |  | 25.4 % |  |
| neutral |  | Observed |  | 59 |  | 74 |  | 133 |  |
|  | | % of total |  | 20.6 % |  | 25.8 % |  | 46.3 % |  |
| least likely |  | Observed |  | 42 |  | 39 |  | 81 |  |
|  | | % of total |  | 14.6 % |  | 13.6 % |  | 28.2 % |  |
| Total |  | Observed |  | 132 |  | 155 |  | 287 |  |
|  | | % of total |  | 46.0 % |  | 54.0 % |  | 100.0 % |  |
|  | | | | | | | | | |

| χ² Tests | | | | | | | |
| --- | --- | --- | --- | --- | --- | --- | --- |
|  |  |  |  |  |  |  |  |
|  | | **Value** | | **df** | | **p** | |
| χ² |  | 1.63 |  | 2 |  | 0.443 |  |
| N |  | 287 |  |  | |  | |
|  | | | | | | | |

# Contingency Tables - BARRIERS

| Contingency Tables | | | | | | | | | |
| --- | --- | --- | --- | --- | --- | --- | --- | --- | --- |
|  | | | | **Q3** | | | |  | |
| **MOT 21 BARRIERS** | |  | | **BSC NURSING** | | **GNM** | | **Total** | |
| most likely |  | Observed |  | 34 |  | 45 |  | 79 |  |
|  | | % of total |  | 11.9 % |  | 15.8 % |  | 27.7 % |  |
| neutral |  | Observed |  | 65 |  | 79 |  | 144 |  |
|  | | % of total |  | 22.8 % |  | 27.7 % |  | 50.5 % |  |
| least likely |  | Observed |  | 29 |  | 33 |  | 62 |  |
|  | | % of total |  | 10.2 % |  | 11.6 % |  | 21.8 % |  |
| Total |  | Observed |  | 128 |  | 157 |  | 285 |  |
|  | | % of total |  | 44.9 % |  | 55.1 % |  | 100.0 % |  |
|  | | | | | | | | | |

| χ² Tests | | | | | | | |
| --- | --- | --- | --- | --- | --- | --- | --- |
|  |  |  |  |  |  |  |  |
|  | | **Value** | | **df** | | **p** | |
| χ² |  | 0.202 |  | 2 |  | 0.904 |  |
| N |  | 285 |  |  | |  | |
|  | | | | | | | |

**Chi square (q18)- OTHERS**

| Contingency Tables | | | | | | | | | |
| --- | --- | --- | --- | --- | --- | --- | --- | --- | --- |
|  | | | | **Q3** | | | |  | |
| **OTHERS** | |  | | **BSC NURSING** | | **GNM** | | **Total** | |
| most likely |  | Observed |  | 30 |  | 34 |  | 64 |  |
|  | | % of total |  | 15.2 % |  | 17.2 % |  | 32.3 % |  |
| neutral |  | Observed |  | 44 |  | 67 |  | 111 |  |
|  | | % of total |  | 22.2 % |  | 33.8 % |  | 56.1 % |  |
| least likely |  | Observed |  | 12 |  | 11 |  | 23 |  |
|  | | % of total |  | 6.1 % |  | 5.6 % |  | 11.6 % |  |
| Total |  | Observed |  | 86 |  | 112 |  | 198 |  |
|  | | % of total |  | 43.4 % |  | 56.6 % |  | 100.0 % |  |
|  | | | | | | | | | |

| χ² Tests | | | | | | | |
| --- | --- | --- | --- | --- | --- | --- | --- |
|  |  |  |  |  |  |  |  |
|  | | **Value** | | **df** | | **p** | |
| χ² |  | 1.67 |  | 2 |  | 0.433 |  |
| N |  | 198 |  |  | |  | |
|  | | | | | | | |

**One-Way ANOVA (Non-parametric) - Q19**

my patients with cancer are generally uninterested in PA

| Kruskal-Wallis | | | | | | | | | |
| --- | --- | --- | --- | --- | --- | --- | --- | --- | --- |
|  |  |  |  |  |  |  |  |  |  |
|  | | **χ²** | | **df** | | **p** | | **ε²** | |
| MOT 19 |  | 0.00155 |  | 1 |  | 0.969 |  | 4.12e-6 |  |
|  | | | | | | | | | |

**Q24 Physical Activity frequencies**

| Descriptive | | | | | | | |
| --- | --- | --- | --- | --- | --- | --- | --- |
|  |  |  |  |  |  |  |  |
|  | | **24 PA** | | **24 PA (2)** | | **24 EVE PA** | |
| N |  | 376 |  | 379 |  | 377 |  |
| Missing |  | 12 |  | 9 |  | 11 |  |
|  | | | | | | | |

**Frequencies**

| Frequencies of 24 PA | | | | | | | |
| --- | --- | --- | --- | --- | --- | --- | --- |
|  |  |  |  |  |  |  |  |
| **Levels** | | **Counts** | | **% of Total** | | **Cumulative %** | |
| 1 |  | 59 |  | 15.7 % |  | 15.7 % |  |
| 2 |  | 78 |  | 20.7 % |  | 36.4 % |  |
| 3 |  | 220 |  | 58.5 % |  | 94.9 % |  |
| 4 |  | 19 |  | 5.1 % |  | 100.0 % |  |
|  | | | | | | | |

| Frequencies of 24 PA (2) | | | | | | | |
| --- | --- | --- | --- | --- | --- | --- | --- |
|  |  |  |  |  |  |  |  |
| **Levels** | | **Counts** | | **% of Total** | | **Cumulative %** | |
| 1 |  | 26 |  | 6.9 % |  | 6.9 % |  |
| 2 |  | 54 |  | 14.2 % |  | 21.1 % |  |
| 3 |  | 260 |  | 68.6 % |  | 89.7 % |  |
| 4 |  | 39 |  | 10.3 % |  | 100.0 % |  |
|  | | | | | | | |

| Frequencies of 24 EVE PA | | | | | | | |
| --- | --- | --- | --- | --- | --- | --- | --- |
|  |  |  |  |  |  |  |  |
| **Levels** | | **Counts** | | **% of Total** | | **Cumulative %** | |
| 1 |  | 36 |  | 9.5 % |  | 9.5 % |  |
| 2 |  | 50 |  | 13.3 % |  | 22.8 % |  |
| 3 |  | 249 |  | 66.0 % |  | 88.9 % |  |
| 4 |  | 42 |  | 11.1 % |  | 100.0 % |  |
|  | | | | | | | |

# One-Way ANOVA (Non-parametric) Q24 - PA

from the statements below tick the best option that describes your response

| Kruskal-Wallis | | | | | | | | | |
| --- | --- | --- | --- | --- | --- | --- | --- | --- | --- |
|  |  |  |  |  |  |  |  |  |  |
|  | | **χ²** | | **df** | | **p** | | **ε²** | |
| 24 PA |  | 3.013 |  | 1 |  | 0.083 |  | 0.00810 |  |
| 24 PA (2) |  | 0.465 |  | 1 |  | 0.495 |  | 0.00124 |  |
| 24 EVE PA |  | 2.323 |  | 1 |  | 0.127 |  | 0.00623 |  |
|  | | | | | | | | | |
