# Supplementary material for: Indian nurses’ beliefs on physical activity promotion practices for cancer survivors in a tertiary care hospital—a cross-sectional survey
Source: PeerJ. 2022 May 23;10:e13348. doi: 10.7717/peerj.13348 (PMC9135035; doi:10.7717/peerj.13348)
Supplement: Supplemental Information 3 [file peerj-10-13348-s003.docx]

**INDIAN NURSES PHYSICAL ACTIVITY PROMOTION PRACTICES AND BELIEFS FOR CANCER SURVIVORS IN A TERTIARY CARE HOSPITAL – A CROSS-SECTIONAL SURVEY.**

**Demographic data:**

1. Age
2. Gender

Male

Female

1. Please list all PROFESSIONAL QUALIFICATIONS which you currently hold
2. Years of practice
3. Years of practice in cancer/tumor group
4. Is the hospital/practice where you work public/private?
5. Where is the hospital/practice located? (Tick the appropriate response)

| Metro |  |
| --- | --- |
| Regional |  |
| Rural |  |

**PREVALENCE OF HEALTH PROMOTION**

1. At your hospital/practice who is the primary person responsible for promoting PHYSICAL ACTIVITY to your patients with cancer? (Tick the appropriate response)

| - Me | - Physiotherapist |
| --- | --- |
| - Oncologist | - **Exercise Physiologist** |
| - Nutritionist/Dietician | - **Don’t know** |
| - Others (please specify) |  |

1. Please indicate the stage(s) at which PHYSICAL ACTIVITY is promoted (tick all that apply)

| Pre-treatment | Post-treatment |
| --- | --- |
| During treatment | **Don’t know** |

1. At your hospital/practice who is the primary person responsible for promoting HEALTHY EATING to your patients with cancer? (Tick the appropriate response)

| - Me | - Physiotherapist |
| --- | --- |
| - Oncologist | - **Exercise physiologist** |
| - Nutritionist/Dietician | - **Don’t know** |
| - Others (please specify) |  |
|  |  |

1. Please indicate the stage(s) at which HEALTHY EATING is promoted (tick all that apply)

| Pre-treatment | Post-treatment |
| --- | --- |
| During treatment | **Don’t know** |

Please select the answers that best describe you

1. I am regularly physically active as defined as -

| - 5-to-30-minute sessions of moderate intensity of exercise |
| --- |
| - 3 or more-minute of sessions of high intensity exercise |
| - I am not regularly physically active |

1. I eat healthy on a regular basis

| - Yes |
| --- |
| - No |

**When is Healthy living promotion the best?**

1. In your opinion when is the best time during the treatment process to promote PHYSICAL ACTIVITY in patients with cancer? with 1 being the most important and 3 being least important. (Tick the appropriate response)

|  | 1 | 2 | 3 |
| --- | --- | --- | --- |
| Pre-treatment |  |  |  |
| During treatment |  |  |  |
| Post-treatment |  |  |  |

1. In your opinion when is the best time during the treatment process to promote HEALTHY EATING in patients with cancer? With 1 being the most important and 3 being least important. (Tick the appropriate response)

|  | 1 | 2 | 3 |
| --- | --- | --- | --- |
| Pre-treatment |  |  |  |
| During treatment |  |  |  |
| Post-treatment |  |  |  |

**MOTIVATION**

1. What benefits may PHYSICAL ACTIVITY have for your patients with cancer? (Tick the appropriate response)

|  | Strongly Disagree | Disagree | Agree | Strongly Agree |
| --- | --- | --- | --- | --- |
| Improves health related quality of life |  |  |  |  |
| Improves weight management |  |  |  |  |
| Improves fatigue level |  |  |  |  |
| Improves mental health |  |  |  |  |
| Improves activities of daily living |  |  |  |  |
| Reduces risk of cancer recurrence |  |  |  |  |
| Reduces the risk of other chronic diseases |  |  |  |  |
| Reduces tumor specific comorbidities |  |  |  |  |
| No benefits |  |  |  |  |

1. What benefits may HEALTHY EATING have for your patients with cancer? (Tick the appropriate response)

|  | Strongly Disagree | Disagree | Agree | Strongly Agree |
| --- | --- | --- | --- | --- |
| Improves health related quality of life |  |  |  |  |
| Improves weight management |  |  |  |  |
| Improves fatigue level |  |  |  |  |
| Improves mental health |  |  |  |  |
| Improves activities of daily living |  |  |  |  |
| Reduces risk of cancer recurrence |  |  |  |  |
| Reduces the risk of other chronic diseases |  |  |  |  |
| Reduces tumor specific comorbidities |  |  |  |  |
| No benefits |  |  |  |  |

1. From the list of factors below, please indicate the THREE most likely to prevent you from promoting PHYSICAL ACTIVITY to your patients with cancer (with 1 being the most likely and 3 being the least likely) (Tick the appropriate response)

|  | 1 | 2 | 3 |
| --- | --- | --- | --- |
| Lack of time |  |  |  |
| Risk to patient |  |  |  |
| Lack of adequate support structure |  |  |  |
| Lack of knowledge |  |  |  |
| Lack of expertise |  |  |  |
| I do not promote Physical activity |  |  |  |
| Not my job |  |  |  |
| I do not have barriers in promoting physical activity |  |  |  |
| Others (please specify) |  |  |  |

1. My patients with cancer are generally uninterested in physical activity (Tick the appropriate response)

| Strongly Disagree | Disagree | Agree | Strongly Agree |
| --- | --- | --- | --- |
|  |  |  |  |

1. Did you face any barriers while promoting physical activity among patients with cancer? (Tick the appropriate response)

| YES |  |
| --- | --- |
| NO |  |

**Healthy Eating Promotion Barriers**

1. From the list of factors below, please indicate the THREE most likely to prevent you from promoting HEALTHY EATING to your patients with cancer (with 1 being the most likely and 3 being the least likely) (Tick the appropriate response)

|  | 1 | 2 | 3 |
| --- | --- | --- | --- |
| Lack of time |  |  |  |
| Risk to patient |  |  |  |
| Lack of adequate support structure |  |  |  |
| Lack of knowledge |  |  |  |
| Lack of expertise |  |  |  |
| I do not promote healthy eating |  |  |  |
| Not my job |  |  |  |
| I have barriers in promoting healthy eating |  |  |  |
| Others (please specify) |  |  |  |

1. My patients with cancer are generally uninterested in healthy eating (Tick the appropriate response)

| Strongly Disagree | Disagree | Agree | Strongly Agree |
| --- | --- | --- | --- |
|  |  |  |  |

1. Did you face any barriers while promoting healthy eating in patients with cancer? If YES identify them. (Tick the appropriate response)

| YES |  |
| --- | --- |
| NO |  |

**DETERMINANTS OF HEALTHY LIVING PROMOTION**

1. From the statements below, please select the best option that describes your response (Tick the appropriate response)

|  | Strongly Disagree | Disagree | Agree | Strongly Agree |
| --- | --- | --- | --- | --- |
| Generally speaking, whether or not I promote Physical Activity to my patients with cancer is entirely up to me |  |  |  |  |
| Generally speaking, whether or not I promote HEALTHY EATING to my patients with cancer is entirely up to me |  |  |  |  |
| My fellow nurses believe I should be promoting PHYSICAL ACTIVITY to my patients with cancer |  |  |  |  |
| My fellow nurses believe I should be promoting HEALTHY EATING to my patients with cancer |  |  |  |  |
| There is a strong evidence base suggesting I should promote PHYSICAL ACTIVITY to my patients with cancer |  |  |  |  |
| There is a strong evidence base suggesting I should promote HEALTHY EATING to my cancer patients |  |  |  |  |
