# Supplementary material for: Indian nurses’ beliefs on physical activity promotion practices for cancer survivors in a tertiary care hospital—a cross-sectional survey
Source: PeerJ. 2022 May 23;10:e13348. doi: 10.7717/peerj.13348 (PMC9135035; doi:10.7717/peerj.13348)
Supplement: Supplemental Information 4 [file peerj-10-13348-s004.docx]

**Mann-Whitney U-Test Table 5**

| Independent Samples T-Test | | | | | | | |
| --- | --- | --- | --- | --- | --- | --- | --- |
|  |  |  |  |  |  |  |  |
|  | |  | | **Statistic** | | **p** | |
| 16 HRQL |  | Mann-Whitney U |  | 17624 |  | 0.620 |  |
| 16 WT |  | Mann-Whitney U |  | 17783 |  | 0.834 |  |
| 16 FATIGUE |  | Mann-Whitney U |  | 17753 |  | 0.894 |  |
| 16 MENTAL |  | Mann-Whitney U |  | 15254 |  | 0.006 |  |
| 16 ADL |  | Mann-Whitney U |  | 16406 |  | 0.079 |  |
| 16 CA |  | Mann-Whitney U |  | 16295 |  | 0.168 |  |
| 16 CHRONIC |  | Mann-Whitney U |  | 17551 |  | 0.981 |  |
| 16 TUMOR |  | Mann-Whitney U |  | 13635 |  | 0.612 |  |
| 16 NO B |  | Mann-Whitney U |  | 8822 |  | 0.027 |  |
| MOT 19 |  | Mann-Whitney U |  | 17638 |  | 0.969 |  |
| 24 PA |  | Mann-Whitney U |  | 15698 |  | 0.083 |  |
| 24 PA (2) |  | Mann-Whitney U |  | 16985 |  | 0.496 |  |
| 24 EVE PA |  | Mann-Whitney U |  | 16083 |  | 0.128 |  |
|  | | | | | | | |

# Table 4 Mean and SD

| Descriptives | | | | | | | | | | | | | | | | | | | | | | | | | | | | | | | | | | | | | | | | |
| --- | --- | --- | --- | --- | --- | --- | --- | --- | --- | --- | --- | --- | --- | --- | --- | --- | --- | --- | --- | --- | --- | --- | --- | --- | --- | --- | --- | --- | --- | --- | --- | --- | --- | --- | --- | --- | --- | --- | --- | --- |
|  |  |  |  |  |  |  |  |  |  |  |  |  | |  |  | |  |  | |  |  | |  |  | |  |  | |  |  | |  |  | |  |  | |  |  |  |
|  | | **Q3** | | **16 HRQL** | | **16 WT** | | **16 FATIGUE** | | **16 MENTAL** | | | **16 ADL** | | | **16 CA** | | | **16 CHRONIC** | | | **16 TUMOR** | | | **16 NO B** | | | **24 PA** | | | **24 PA (2)** | | | **24 EVE PA** | | | **MOT 19** | | |  |
| Mean |  | 1 |  | 2.97 |  | 2.94 |  | 2.88 |  | 3.13 |  | 2.99 | |  | 2.77 | |  | 2.89 | |  | 2.70 | |  | 1.92 | |  | 2.45 | |  | 2.79 | |  | 2.71 | |  | 2.50 | |  |  |  |
|  |  | 2 |  | 2.98 |  | 2.98 |  | 2.94 |  | 3.00 |  | 2.93 | |  | 2.89 | |  | 2.92 | |  | 2.72 | |  | 2.14 | |  | 2.59 | |  | 2.85 | |  | 2.85 | |  | 2.51 | |  |  |  |
| Standard deviation |  | 1 |  | 0.853 |  | 0.813 |  | 0.937 |  | 0.862 |  | 0.843 | |  | 0.889 | |  | 0.883 | |  | 0.848 | |  | 0.954 | |  | 0.865 | |  | 0.742 | |  | 0.836 | |  | 0.780 | |  |  |  |
|  |  | 2 |  | 0.734 |  | 0.567 |  | 0.651 |  | 0.671 |  | 0.641 | |  | 0.669 | |  | 0.701 | |  | 0.783 | |  | 0.883 | |  | 0.771 | |  | 0.667 | |  | 0.694 | |  | 0.693 | |  |  |  |
|  | | | | | | | | | | | | | | | | | | | | | | | | | | | | | | | | | | | | | | | | |
